# ProfileGrids: a sequence alignment visualization paradigm that avoids the limitations of Sequence Logos

**DOI:** 10.1186/1753-6561-8-S2-S6

**Published:** 2014-08-28

**Authors:** Alberto I Roca

**Affiliations:** 1 ProfileGrid.org, P.O. Box 6414, Irvine, California 92616, USA

## Abstract

**Background:**

The 2013 BioVis Contest provided an opportunity to evaluate different paradigms for visualizing protein multiple sequence alignments. Such data sets are becoming extremely large and thus taxing current visualization paradigms. Sequence Logos represent consensus sequences but have limitations for protein alignments. As an alternative, ProfileGrids are a new protein sequence alignment visualization paradigm that represents an alignment as a color-coded matrix of the residue frequency occurring at every homologous position in the aligned protein family.

**Results:**

The JProfileGrid software program was used to analyze the BioVis contest data sets to generate figures for comparison with the Sequence Logo reference images.

**Conclusions:**

The ProfileGrid representation allows for the clear and effective analysis of protein multiple sequence alignments. This includes both a general overview of the conservation and diversity sequence patterns as well as the interactive ability to query the details of the protein residue distributions in the alignment. The JProfileGrid software is free and available from http://www.ProfileGrid.org.

## Background

Molecular biologists can learn about a protein's structure and function by studying the natural sequence variation resulting from a protein family's evolution [1]. A multiple sequence alignment (MSA) is crucial for such bioinformatic analysis to relate homologous residues to one another as pioneered by early molecular evolution studies (for example [2]). Software tools are now available to align very large data sets consisting of 100,000+ sequences [3]. However, there is a lack of programs that allow users to visualize and to interact with very large MSAs [4]. Existing MSA visualization tools [5] continue to use a simple stacked sequence alignment visualization paradigm representing all individual sequences as rows in a table and homologous residue positions as columns. This "row-column" paradigm was sufficient decades ago when alignments were small; but, the stacked sequence representation is now inadequate for the amount of data being used today.

Sequence Logos were introduced in 1990 as a new visualization paradigm to represent the MSA consensus residues enabling easy observation of conservation patterns in MSAs [6]. A hallmark of this paradigm is that a residue's frequency in the MSA column is depicted visually by the height of the residue single-letter symbol with all residues from the same column then stacked upon each other. These features are shared by subsequent derivatives of the Sequence Logo paradigm (Table 1). Modifications to the paradigm include different algorithms for determining symbol height [7-16], visualizing conservation within MSA subpopulations [17,18], replacing the symbols with one-dimensional [19] or three-dimensional [20] bars, comparing differences between two MSAs [11], identifying infrequent residues [15,21], handling specialized contexts (such as for RNA [7,22,23], structural elements [24], or codons [25]), and implementing online web servers [26].

**Table 1:** Sequence Logo Derivatives

Name	Year	Reference
SequenceLogo	1990	6
StructureLogo	1997	7
WebLogo	2004	26
HMMLogo	2004	8
PSSMlogo	2004	9
enoLOGOS	2005	10
CorreLogo	2006	20
InverseLogo	2006	21
LogoBar	2006	19
SubfamilyLogo	2006	17
TwoSampleLogo	2006	11
Phylo-mLogo	2007	18
REALLogo	2007	24
Blogo	2008	12
RNALogo	2008	22
iceLogo	2009	13
berryLogo	2011	14
CodonLogo	2012	25
Rilogo	2012	23
Seq2Logo	2012	15
pLogo	2013	16
*BioVis contestants*	2013	(*this volume*)

Despite their widespread use, though, Sequence Logos have their limitations. While Logos are suitable for nucleic acids with only four residue symbols legible at even small sizes, there are problems when representing 20 residues of protein sequences. Variable regions of a protein alignment and positions with rare residues are not legible in Logos since the symbol size is small when scaled relative to the conserved positions. This has been described as a "totally incomprehensible jumble of letters." [14] However, even for conserved residues, the stacking of the symbols can lead to confusion. For example, Schneider pointed out that an "F" on top of an "L" could be mistaken for a long "E." [6] Logos do not display any information about residues missing from alignment columns and also lack a representation for gap symbols. Notably, Logos suffer from aesthetic challenges when two visualization channel types [27] are combined: symbol/stack height versus symbol color. Interpreting the tall/short stacked letter columns can be complicated by the color schemes used to distinguish different amino acid residue classes (such as the prominent red coloring of acidic protein residues). Finally, a recent user study evaluated the effectiveness of Sequence Logos for comparing motifs [28]. The authors identified these problems: difficulty in judging the height of stacked symbols, no standardization for symbol color schemes, and a lack of interactivity for most current software tools.

The challenge in the visualization of large sequence alignments is in identifying a paradigm that summarizes the overall conservation trends while still providing easy navigation to detailed views of the underlying data [4]. ProfileGrids as a MSA visualization paradigm were invented in 2005 for the analysis of the bacterial RecA protein family. MSA analysis had become impractical using the standard stacked sequence representation since a curated alignment had grown to several hundred homologs [29]. A ProfileGrid reduces an alignment to a matrix, color-shaded according to the residue frequency in the MSA [30]. The JProfileGrid Java program was upgraded to version 2.0 [31] with new software features as well as improvements to the aesthetics in the visualization paradigm after receiving feedback from the http://VIZBI.org community [27]. The ProfileGrid paradigm has two simple but significant differences that avoid Sequence Logos limitations: 1) all residue symbols are the same height since the matrix row sizes are fixed; 2) color shading of the cells is used to represent the frequency distribution of the residues in the sequence data. Thus, the overall conservation trends can be seen from the "heat map." The JProfileGrid software tool is an interactive MSA viewer taking advantage of the matrix representation of alignments.

The 2013 BioVis conference Redesign Contest provided an opportunity to demonstrate the usefulness of the ProfileGrid paradigm by visualizing the adenylate kinase lid (AKL) protein family alignments [32]. Here, I report the final figures generated by the JProfileGrid software and the unique observations made possible by ProfileGrid analysis.

## Methods

**Figure 1:**
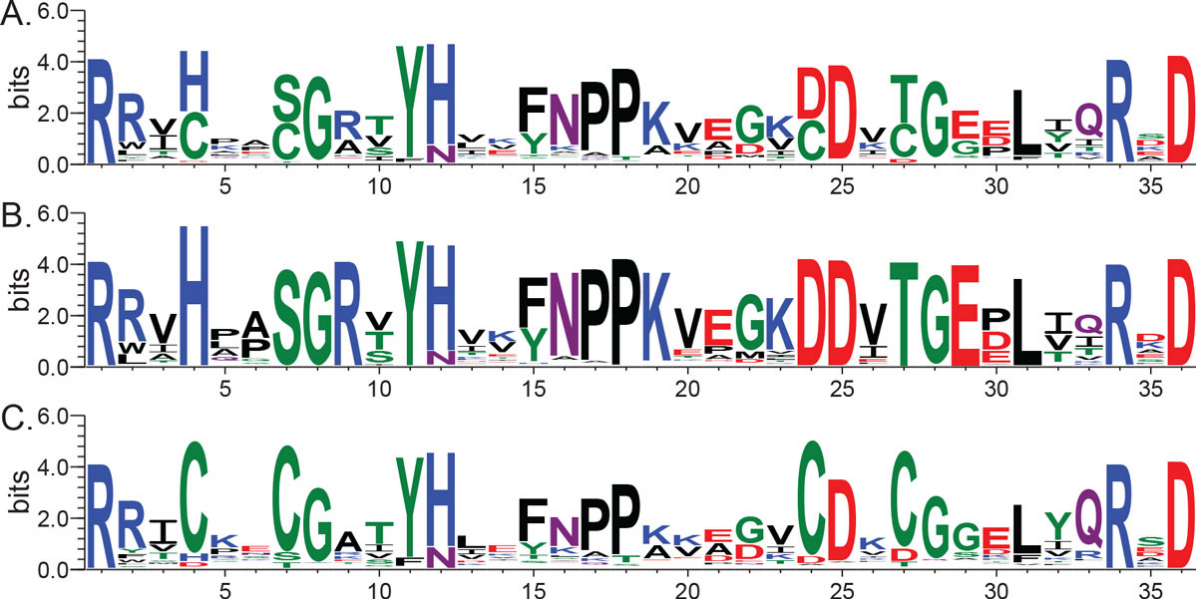
**Sequence Logo visualization of the AKL alignments**. Sequence Logos of the adenylate kinase lid (AKL) domain multiple sequence alignments (A) across all organisms for 1,809 protein sequences, (B) from Gram negative bacteria for 923 sequences, and (C) from Gram positive bacteria for 886 sequences.

The protein sequence alignment data sets were provided by the 2013 BioVis Redesign Contest organizers. The Sequence Logos (Figure 1) were generated using the WebLogo server [26] and replicate the original BioVis contest figure to be "redesigned." Two versions of the JProfileGrid software, 1.22 [30] and 2.0.5 [31], have been described in previous publications. The latter version was used for this study and is available under a GNU General Public License at http://www.ProfileGrid.org.

## Results

### ProfileGrids clearly visualize protein residue distributions

The 2013 BioVis Redesign Contest description "recognizes the importance of effective encodings and clear visual communication in display of complex quantitative information" and "gives participants the opportunity to develop a practical replacement to the long-standing convention of sequence logos" [32] as exemplified by the AKL domain Sequence Logos (Figure 1). ProfileGrids are a practical and effective replacement for Sequence Logos when analyzing protein alignments. Furthermore, the mature JProfileGrid MSA software viewer has a rich graphical user interface allowing a molecular biologist to analyze their protein family of interest [30].

**Figure 2:**
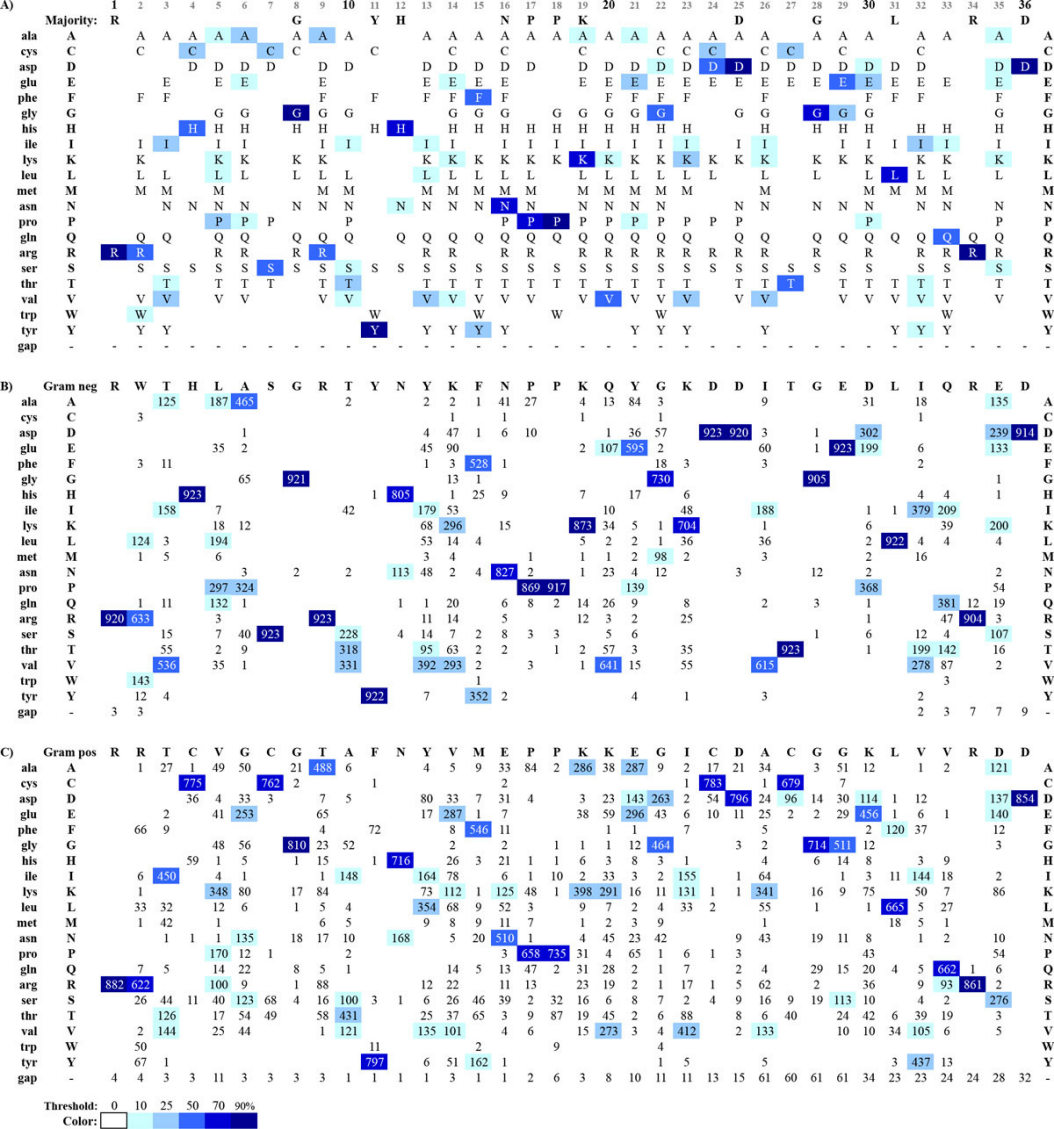
**ProfileGrid visualization of the AKL alignment protein residue distributions**. ProfileGrids showing the amino acid usage in the adenylate kinase lid (AKL) domain (A) across all organisms for 1,809 sequences, (B) from Gram negative bacteria for 923 sequences, and (C) from Gram positive bacteria for 886 sequences. The ProfileGrid panels show a representative sequence from the Gram negative (B) and Gram positive (C) alignments while panel (A) shows a majority consensus. For each panel, the color shading is normalized to the number of sequences in each alignment as a color ramp from white (<10%) to dark blue (>= 90% conservation). This figure was exported from the JProfileGrid software and takes advantage of many user defined options such as representing the residue symbols (A) versus the frequency counts (B, C).

The AKL domain sequence alignments were imported into JProfileGrid for visualization and analysis to determine the user settings for the final figures. Two visualizations were designed to demonstrate the ProfileGrid paradigm's capabilities as well as to represent observations from the AKL domain MSAs. The entire alignment of 1,809 sequences is shown in Figure 2A with the default JProfileGrid settings where the 20 protein residue rows are sorted alphabetically (with one additional gap symbol row). The frequency of the residues in each alignment is represented by color shades according to a blue ramp from low (white) to high (dark blue) conservation across the 36 residue width (columns) of the AKL sequences. The lower panels show the subpopulations for the 923 sequences from Gram negative bacteria (Figure 2B) and the 886 sequences from Gram positive bacteria (Figure 2C). The latter two panels show a single representative reference sequence at the top of the ProfileGrid for each respective subpopulation.

Overall trends of sequence conservation and variability are quite clear from the ProfileGrids and the user can choose whether to show the details of the residue frequencies (values in panel Figures 2B and 2C) or to show just the residues observed (Figure 2A) at that MSA position, *i.e*. each column. Note that the ProfileGrid in Figure 2A can be directly related to the Sequence Logo (Figure 1A) where the residues shown are stacked upon one another in each column; and, the symbol height reflects the conservation of each residue. The ProfileGrid, however, allows each symbol to be legible which is critical for interpreting a variable column such as position 14. The ProfileGrid allows the entire protein mutation distribution to be assessed. Similarly, rare residues are obvious such as at position 8 (Figure 2B) where the Redesign Contest instructions incorrectly stated that "glycine is absolutely conserved in the Gram-negatives." [33] In fact, 2 sequences have asparagine at that position (*Theileria parva *strain Muguga [GenBank:XP_766154.1 http://www.ncbi.nlm.nih.gov/protein/71033025] and *Theileria annulata *[Genbank:XP_954152.1 http://www.ncbi.nlm.nih.gov/protein/XP_954152.1]). Such rare observations would be impossible to identify from the Sequence Logo paradigm (Figure 1B) or its derivatives (the exception being InverseLogos depending upon user settings [21]). An AKL protein expert can now interpret whether the Gram negative homologs containing 8-asn are interesting natural sequence variation exceptions sharing a residue with 18 Gram positive homologs (Figure 2C) or are just sequence errors resulting from experimental problems during data collection [30]). The interactive JProfileGrid user interface made it trivial to identify the two asparagine-containing sequences since the user can select any cell in the ProfileGrid window and then perform a query of the MSA. Thus, the ProfileGrid paradigm enables the MSA to be treated as a searchable database of sequence records.

### ProfileGrids can effectively visualize alignment differences

The 2013 BioVis Redesign Contest challenged applicants to help the AKL biologists understand the differences between the Gram negative (Figure 1B) and Gram positive (Figure 1C) sequences by creating alternatives to the Sequence Logo representation. Visualizing the protein sequence differences between the two subpopulations would presumably allow the biologists to propose structure and function hypotheses about the AKL protein activities. The original figure designed by the AKL biologists is of three stacked Sequence Logos (Figure 1). Drawing comparisons between protein Sequence Logos is very difficult especially since there is no visual encoding for differences (although attempts have been made to introduce such a feature [11,17,18]). In particular, a user will be frustrated performing mental comparisons on adjacent Sequence Logos such as when comparing the Gram negative (Figure 1B) and Gram positive (Figure 1C) sequences. Such challenges were documented in a recent Sequence Logo user study [28]. By contrast, the interactive JProfileGrid software is especially suited for comparing sequences populations to one another.

**Figure 3:**
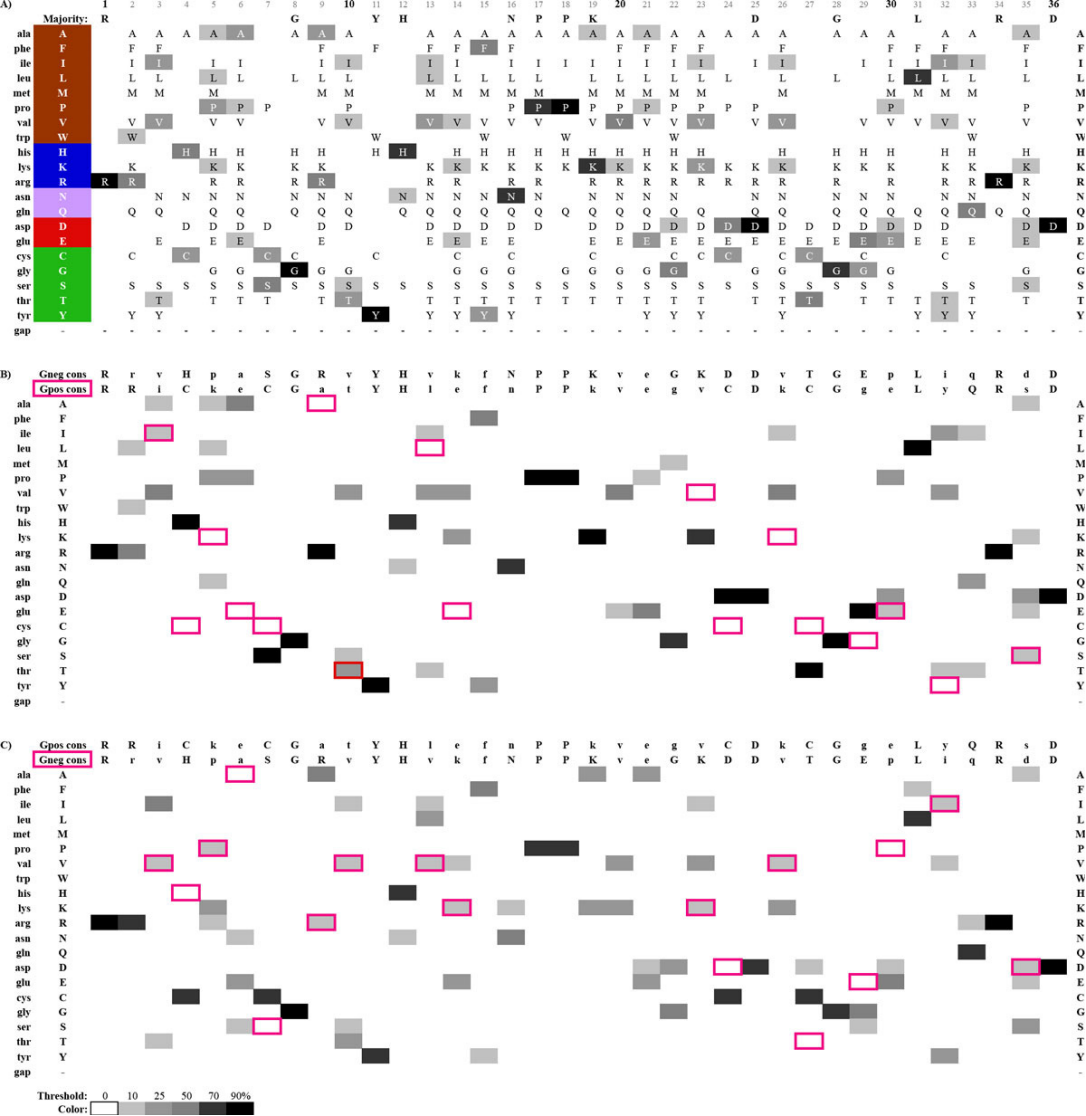
**ProfileGrids highlighting differences between AKL alignment subpopulations**. The JProfileGrid software offers many user defined options to customize the appearance of the ProfileGrid for final figure export here shown for the AKL alignment across all organisms for 1,809 sequences (A), from Gram negative bacteria for 923 sequences (B), and from Gram positive bacteria for 886 sequences (C). The ProfileGrid panel first row shows the consensus sequence from the Gram negative (B) and Gram positive (C) alignments while panel (A) shows the majority residues from the entire MSA. The pink boxes in panels (B) and (C) highlight differences between the two consensus sequences shown. For each panel, the color shading is normalized to the number of sequences in each alignment as a grayscale ramp from white (<10%) to black (>= 90% conservation). For clarity, the ProfileGrid cells in panels (B) and (C) do not show any values or symbols to facilitate visual inspection of the patterns. The residue codes in the panel (A) second column are color shaded to represent the following amino acid classes: hydrophobic (brown), basic (blue), nitrogen-containing (lavender), acidic (red), and other (green). The ProfileGrid rows are sorted according to the amino acid classes whereas in Figure 2 the rows are sorted alphabetically by residue symbol.

ProfileGrids can effectively highlight differences between the AKL data sets (Figure 3). For this purpose, we calculated a consensus sequence for each subpopulation and list them both at the top of each subpopulation ProfileGrid (Figure 3B & 3C) where the first row is the "reference" and the second row is the "highlight" sequence to be compared. For each column, if the highlight sequence differs from the reference, then the residue cell is marked with a pink border automatically generated by the JProfileGrid software [30]. Viewing the "highlight boxes" within the context of the entire reference subpopulation alignment clearly shows the differences between the Gram negatives (Figure 3B) and Gram positives (Figure 3C) since the highlight consensus acts as a proxy for its entire alignment. For the sake of visual clarity, the residue frequency shading is grayscale (instead of the default blue ramp) and the values are not shown in the cells.

The ProfileGrid paradigm can allow color coding to visually represent amino acid classes as used in Sequence Logos. However, in this case the coloring is minimized to provide just enough information rather than dominate the figure and confuse the user. Figure 3A shows a color scheme to represent hydrophobic (brown), basic (blue), nitrogen-containing (lavender), acidic (red), and other (green) residues. Note that brown is used instead of black (as in the default AKL Sequence Logo coloring of Figure 1) to avoid visually clashing with the frequency grayscale encoding. Thus, as a reader scans across the ProfileGrid, the row position will encode the amino acid class in a manner complementary to the frequency information.

The ProfileGrid highlight (pink cell border) feature takes advantage of "visual popout" [27] to allow the user to make observations. AKL alignment position 9 is a good case study to examine differences between the Gram negative and Gram positive subpopulations. In Figure 3B arginine (positively charged coded "blue," middle rows as per Figure 3A) is very well conserved (black cell; row 13, column labelled "9") in Gram negatives. However, an alanine (Figure 3B; pink borders; row 3, column labelled "9") is found in Gram positives and this residue is small and hydrophobic (brown, top rows). Intriguingly, examination of the Gram positive sequences (Figure 3C) shows that there are at least two predominant subpopulations observed (see Figure 2C for the complete residue distribution at position 9): the aforementioned alanine (gray box; row 3, column 9) but also the arginine (gray box with pink borders; row 13, column 9) shared with the Gram negatives. Perhaps the AKL experts can determine if there is any biological significance to these qualitative observations; but, this shows that the ProfileGrid paradigm allows these observations to be made at all.

### JProfileGrid program features

The interactive JProfileGrid program viewer has features designed for the biologist user [30,31]. In software version 2.0, a new "overview" mode allows the visualization of the entire MSA within one window as either a ProfileGrid or as stacked sequences. Individual ProfileGrid cells can be selected to extract sequence subsets of interest during a visualization dissection. Sorting the residue rows by physical/chemical properties such as flexibility, helix propensity, hydropathy, and volume allow qualitative structural analyses to be performed. The detailed ProfileGrid window with the symbol counts, has a new second pane to view different parts of the MSA at the same time. The "highlight" feature can identify residues that occur greater or less than a user-defined threshold of residue frequency. Large alignments can be separated into subsets of interest by using metadata filtering once JProfileGrid imports simple sequence annotations from flat file spreadsheet databases. The interactive features of the JProfileGrid program can be more easily appreciated by a live walk-through as demonstrated by my 2013 BioVis Data Contest movie submission [34].

### ProfileGrid advantages over Sequence Logos/Bundles

ProfileGrids avoid the limitations of Sequence Logos especially for protein MSAs. Namely, all ProfileGrid residue symbols are legible so that no information is lost for bioinformatic analysis. ProfileGrids display gap symbols as well as the regions of a MSA where residues are *not *observed. Neither case is handled by Sequence Logos. The ProfileGrid paradigm, in the opinion of this author, clearly and effectively separates visual encoding channels to be more aesthetically pleasing than Sequence Logos. Such clarity allows for the careful dissection of sequence conservation patterns in MSAs by molecular biologists. Importantly, ProfileGrids solve the visualization problem of handling very large alignments since there is almost no limit to the number of sequences that can be represented. The matrix representing the MSA is only 21 rows rather than the inefficient stacked sequence representation that lists every individual sequence as a row. While a Sequence Logo can also represent very large MSAs, the details of the underlying sequence information are lost as in most consensus paradigms. The interactive JProfileGrid viewer allows the user to retain access to the sequence data for a robust protein family analysis.

Finally, we comment on the Redesign Contest applicants. Most of the submissions were derivatives of Sequence Logos and so would have the same aforementioned limitations. Interestingly, the two entries that received Honorable Mentions [35,36] have replaced the Sequence Logo representation with a different visualization paradigm that one entrant named "Sequence Bundles." We note that by representing the MSA as a matrix with residue symbols as rows and the alignment positions as columns, they are handling the underlying data as a profile for bioinformatic sequence similarity searches [37] using command-line programs with no graphical user interfaces. The 2008 ProfileGrid publication, to the best of my knowledge, is the first demonstration of profiles as a visual paradigm for analyzing MSAs. How Sequence Bundles differ significantly from ProfileGrids is in the former's use of lines to connect matrix cells along the path of an individual protein sequence. More sequences occurring in an alignment result in lines stacking on the matrix visualization allowing consensus sequences to emerge as a thick bundle.

While the Sequence Bundle connecting lines are aesthetically interesting, they are also a liability since as the MSAs get very large, the visualization will become more cluttered. In my opinion, what is relevant to the biologist user within the context of an MSA is the frequency of the residues in the visualization. After an observation has been made, then the user will dig deeper to identify the particular sequences of interest. The interactive JProfileGrid program was designed for such a task. Individual sequences are shown alongside the top row of the ProfileGrid by choosing the desired reference and highlight sequences from JProfileGrid menus. Selecting individual ProfileGrid cells allow a separate JProfileGrid window to display the subset of the sequences containing that residue in the MSA. There is no need for connecting lines to be layered on top of the matrix as in Sequence Bundles.

## Conclusions

While Sequence Logos have their merits for visualizing short conserved motifs especially in nucleic acid sequences, this paradigm has limitations when representing protein alignments. The ProfileGrid paradigm replaces Sequence Logos and solves the challenge of visualizing large protein alignments. Thus, ProfileGrids allow a molecular biologist to clearly and effectively analyze protein structure and function.

## List of abbreviations used

AKL: adenylate kinase lid

MSA: multiple sequence alignment

## Competing interests

The author declares that they have no competing interests.

## Authors' contributions

AIR conceived of the study, conducted the analyses, and authored the manuscript.

## Declarations

Publication of this work was self-financed by AIR.

This article has been published as part of *BMC Proceedings *Volume 8 Supplement 2, 2014: Proceedings of the 3rd Annual Symposium on Biological Data Visualization: Data Analysis and Redesign Contests. The full contents of the supplement are available online at http://www.biomedcentral.com/bmcproc/supplements/8/S2
